# Synthesis and Biological Screening of New 4-Hydroxycoumarin Derivatives and Their Palladium(II) Complexes

**DOI:** 10.1155/2021/8849568

**Published:** 2021-04-28

**Authors:** Edina H. Avdović, Isidora P. Petrović, Milena J. Stevanović, Luciano Saso, Jasmina M. Dimitrić Marković, Nenad D. Filipović, Miroslav Ž. Živić, Tijana N. Cvetić Antić, Milan V. Žižić, Nataša V. Todorović, Milena Vukić, Srećko R. Trifunović, Zoran S. Marković

**Affiliations:** ^1^Department of Science, University of Kragujevac, Institute of Information Technologies, Jovana Cvijica bb, 34000 Kragujevac, Serbia; ^2^Faculty of Science, University of Kragujevac, Radoja Domanovića 12, 34000 Kragujevac, Serbia; ^3^Institute of Molecular Genetics and Genetic Engineering, University of Belgrade, Vojvode Stepe 444a, PO Box 23, 11010 Belgrade, Serbia; ^4^Faculty of Biology, University of Belgrade, Studenski trg 16, 11000 Belgrade, Serbia; ^5^Serbian Academy of Sciences and Arts (SASA), Kneza Mihaila 35, 11000 Belgrade, Serbia; ^6^Department of Physiology and Pharmacology “Vittorio Erspamer”, Sapienza University of Rome, Piazzale Aldo Moro 5, 00185 Roma, Italy; ^7^Faculty of Physical Chemistry, University of Belgrade, Studenski trg 12-16, 11000 Belgrade, Serbia; ^8^Faculty of Engineering, University of Kragujevac, Sestre Janjic 6, 34000 Kragujevac, Serbia; ^9^Institute for Multidisciplinary Research, Department of Life Sciences, University of Belgrade, Kneza Višeslava 1, 11030 Belgrade, Serbia; ^10^Institute for Biological Research “Sinisa Stankovic”, Institute of National Importance for the Republic of Serbia, University of Belgrade, Bulevar Despota Stefana 142, 11000 Belgrade, Serbia

## Abstract

Two newly synthesized 4-hydroxycoumarin bidentate ligands (L1 and L2) and their palladium(II) complexes (C1 and C2) were screened for their biological activities, *in vitro* and *in vivo*. Structures of new compounds were established based on elemental analysis, ^1^H NMR, ^13^C NMR, and IR spectroscopic techniques. The obtained compounds were tested for their antioxidative and cytotoxic activities and results pointed to selective antiradical activity of palladium(II) complexes towards ^•^OH and ^-•^OOH radicals and anti-ABTS (2,2′-Azino-bis(3-ethylbenzothiazoline-6-sulfonic acid) cation radical) activity comparable to that of ascorbate. Results indicated the effect of C1 and C2 on the enzymatic activity of the antioxidative defense system. *In vitro* cytotoxicity assay performed on different carcinoma cell lines (HCT166, A375, and MIA PaCa-2), and one healthy fibroblast cell line (MRC-5) showed a cytotoxic effect of both C1 and C2, expressed as a decrease in carcinoma cells' viability, mostly by induction of apoptosis. *In vivo* toxicity tests performed on zebrafish embryos indicated different effects of C1 and C2, ranging from adverse developmental effect to no toxicity, depending on tested concentration. According to docking studies, both complexes (C1 and C2) showed better inhibitory activity in comparison to other palladium(II) complexes.

## 1. Introduction

Cancer is characterized by uncontrolled cell growth and represents one of the major causes of mortality according to the World Health Organization [1]. Some circumstantial evidence implies that the initiation of carcinogenesis is closely related to the sustained oxidative stress which leads to the production of radical species, especially oxygen and nitrogen ones (ROS, NRS), causing significant damage to cell structure and functions. Carcinogenesis can be mediated by radical species either directly (inducing DNA mutations, nitration, oxidation, mitophagy, etc.) or indirectly by activation of signaling pathways [2]. The conventional approaches as surgery, conventional radiotherapy, or chemotherapy are not completely efficient in most cancer treatments. An additional problem is the increase of drug resistance which can occur through various mechanisms such as modifications in the binding site of the selected target, metabolism, or drug export systems [3, 4]. Side-effects are usually caused by nonspecific drug delivery and include risks of infection, anemia and bleeding, nausea and vomiting, hair loss, gastrointestinal irritation, fatigue, and accelerated menopause in women [5]. Therefore, the development and discovery of novel and effective anticancer drugs are currently a great challenge for researchers worldwide.

In the search for phytochemicals with a broad spectrum of biological activity and low toxicity, coumarins have attracted intense interest from many research groups. Coumarins, oxygen-containing heterocycles with typical benzopyrone scaffold, are naturally occurring compounds isolated from different plants, fungi, and bacteria [6]. This class of phytochemicals shows diverse biological and pharmaceutical effects such as anticoagulant, antifungal, hepatoprotective, antithrombotic, antiviral, antimicrobial, antituberculosis, anticarcinogenic, antidepressant, antihyperlipidemic, anticholinesterase, anti-inflammatory, antinociceptive, and antioxidant activities [1, 3, 7–14]. Some coumarin-based drugs have been extensively used in medicine as anticoagulants (warfarin, acenocoumarol, cyclocumarol, and dicoumarol) and antineurodegenerative agents [10, 12]. Despite numerous biological effects described above, coumarins are considered as one of the most versatile compounds in the design and discovery of anticancer drugs because they provide minimal side effects along with multidrug resistance reversal activity [15]. Literature data show that mechanisms by which coumarin and their derivatives can exert anticancer activity include inhibition of the telomerase or protein kinase activity, downregulation of oncogene expression, induction of the caspase-9 mediated apoptosis, suppression of cell proliferation by arresting cell cycle in G0/G1 and G2/M phases, or through inhibition of P-glycoprotein in cancer cells [1, 16]. Intending to enhance the biological activity of coumarin researchers are opting for the synthesis of the complexes.

It should be emphasized that the complexes of cerium(III), zirconium(IV), copper(II), zinc(II), bismuth(III), and cadmium(II) containing coumarins as ligand have shown *in vitro* cytotoxic activity [17]. Palladium(II) complexes present alternative candidates for antitumor metallo-based drugs due to their structural and thermodynamic resemblances to the platinum(II) complexes which have been widely used in the treatment of various malignancies. Compared to extensively used carboplatin, it is shown that palladium(II) complexes containing coumarins as ligands, where the environment of the palladium atom is similar to that of carboplatin, display 7800 times higher cytotoxicity [17, 18]. Also, compared to platinum(II) complexes, palladium(II) complexes have better solubility and aquation and ligand-exchange rates about 10^4^–10^5^ times greater [18, 19]. Regarding presented advantages, there is growing interest in the synthesis and examination of biological effects of palladium(II)-coumarin-based complexes.

Besides, numerous complexes of ruthenium, gold, and palladium with noncoumarin ligands such as terpyridine, tetrapyridine, and 1,10-phenanthroline, ligands are known to occupy a significant place in cytotoxic research [20–23].

This paper presents the continuation of our work on the design of novel coumarin-based ligands and their corresponding palladium(II) complexes [24–27]. The compounds are tested for their antioxidative, *in vitro*, and *in vivo* cytotoxic effects and the effect on the activity of antioxidative enzymes. The effective binding of novel compounds to receptor tyrosine kinase has been investigated by a molecular docking study.

## 2. Materials and Methods

### 2.1. Substances

*Meta*-aminophenol, *para*-aminophenol, 4-hydroxycoumarin, acetic acid, methanol, ethanol, toluene, acetone, dimethyl sulfoxide, potassium-tetrachloridopalladate(II), and phosphoryl chloride were obtained from Sigma-Aldrich, Germany.

### 2.2. Measurements

Elemental analyses for C, H, and N were performed on a Vario EL III C, H, N Elemental Analyzer. Infrared spectra (KBr) were recorded on a Perkin-Elmer Spectrum One FT-IR spectrometer (4000-400 cm^−1^). ^1^H NMR and ^13^C NMR spectra were recorded in DMSO-d*_6_* on a Varian Gemini 200 spectrometer (Varian, Palo Alto, CA) operating at 200 MHz and 50 MHz, respectively. The chemical shift values were given in *δ* (ppm) units, while coupling constants (*J*) were given in Herz (Hz). TMS was used as the internal standard. The signal profiles determined by neighbor-induced splittings in the reported ^1^H NMR spectra were defined as s-singlet, dd-doublet of a doublet, m-multiplet, and brs-broadened singlet. Analytical TLC was performed on silica gel (Silica gel 60, layer 0.20 mm, Alugram Sil G, Mashery-Nagel, Germany), while the visualization of TLC plates was performed using a UV lamp at 254 nm and 365 nm (VL-4.LC, 365/254, Vilber Lourmat, France).

### 2.3. General Procedure for the Synthesis of Ligands and Palladium(II) Complexes

**Scheme 1:**
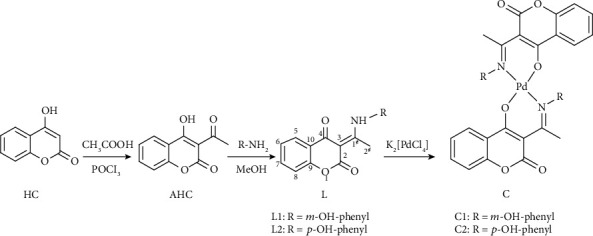
The general procedure for the synthesis of the ligands L1 and L2 and complexes C1 and C2.

Initially, heterocyclic compound 3-acetyl-4-hydroxycoumarin (AHC) was synthesized, as previously described [28], in the reaction of 4-hydroxycoumarin (HC) and acetic acid with phosphoryl chloride as a catalyst. In the next step, the reaction of synthesized AHC with *meta*-hydroxyaniline and *para*-hydroxyaniline in dry methanol resulted in the formation of the final products L1 and L2 with good yields (89% and 86%). The coupling reaction was performed following the previously published synthesis of AHC [24–27]. To obtain compounds with very high purity, the final compounds were further purified by recrystallization from ethanol. The synthesis of two corresponding palladium complexes, C1 and C2, was performed in one step reaction of K_2_[PdCl_4_] and the bidentate ligands L1 and L2 (Scheme 1). Each of the bidentate ligands consisted of an oxygen donor atom, positioned on *α*-pyrone and a nitrogen donor atom of aminophenol. The reaction involved the dropwise addition of methanol solution of bidentate ligands to an aqueous solution of K_2_[PdCl_4_] with continuous stirring for 5 h. After precipitation, filtration, and air drying, the new palladium complexes were obtained in moderately good yields (46-89%).

*3-(1-((3-Hydroxyphenyl)amino)ethylidene)chroman-2,4-dione* (L1). White powder. Yield: 0.64 g (89%). *Anla. calc.* For C_17_H_13_O_4_N (Mr = 295.28) %: C, 69.15; H, 4.44; N, 4.74. Found: C, 68.75; H, 4.24; N, 4.76. IR (KBr) *ν* cm^−1^: 3228 (OH, NH); 3064 (=CH); 2992, 2934, and 2724 (CH); 1675 (C=O); 1607, 1599, 1564, and 1488 (C=C); 1147 (C-O). ^1^H NMR (200 MHz, DMSO-d_6_) *δ* ppm: 2.57 (s, 3H, C–2′), 6.77 (m, 1H, C–6^″^), 6.79 (m, 1H, C–4^″^), 6.87 (m, 1H, C–5^″^), 7.28 (m, 1H, C–6), 7.31 (m, 1H, C–7), 7.35 (s, 1H, C–2^″^), 7.65 (m, 1H, C–5), 7.95 (dd, 1H, ^3^JH_8_, H_7_ = 6 Hz, ^4^JH_8_, H_6_ = 2 Hz, C–8–H), 9.99 (bs, 1H, OH), 15.42 (bs, 1H, NH). ^13^C NMR (50 MHz, DMSO-d_6_) *δ* ppm: 20.6 (C–2′), 97.3 (C–3), 112.5 (C–2^″^), 115.5 (C–4^″^), 116.2 (C–5^″^), 116.5 (C–8), 119.9 (C–5), 124.0 (C–6^″^), 126.0 (C–6), 130.6 (C–10), 134.6 (C–7), 137.0 (C–1^″^), 153.4 (C–3^″^), 158.5 (C–9), 161.7 (C–2), 175.8 (C–4), 180.5 (C–1′).

*3-(1-((4-Hydroxyphenyl)amino)ethylidene)chroman-2,4-dione* (L2). White powder. Yield: 0.62 g (86%). *Anla. calc.* For C_17_H_13_O_4_N (Mr = 295.28) %: C, 69.15; H, 4.44; N, 4.74. Found: C, 68.76; H, 4.37; N, 4.72. IR (KBr) *ν* cm^−1^: 3345 (OH); 3219 (NH); 3068 (=CH); 2934, 2826, and 2696 (CH); 1687 (C=O); 1611, 1564, 1513, and 1484 (C=C); 1198 (C–O). ^1^H NMR (200 MHz, DMSO-d_6_) *δ* ppm: 2.55 (s, 3H, C–2′), 6.89 (m, 2H, C–H–2^″^, C–H–6^″^), 7.20 (m, 2H, C–3^″^–H, C–5^″^–H), 7.28 (m, 1H, C–6–H), 7.30 (m, 1H, C–7–H), 7.64 (m, 1H, C–5–H), 7.94 (dd, 1H, ^3^J H_8_, H_7_ = 12 Hz, ^4^J H_8_, H_6_ = 2.1 Hz, C–8–H), 9.91 (bs, 1H, OH), 15.28 (bs, 1H, NH). ^13^C NMR (50 MHz, DMSO-d_6_) *δ* ppm: 20.5 (C–2′), 97.1 (C–3), 116.2 (C–3^″^, C–5^″^), 116.5 (C–8), 120.0 (C–5), 123.9 (C–2^″^, C–6^″^), 125.9 (C–6), 126.9 (C–10), 127.2 (C–1^″^), 134.5 (C–7), 153.4 (C–4^″^), 157.5 (C–9), 161.8 (C–2), 175.7 (C–4), 180.3 (C–1′).

*Bis(3-(1-((3-hydroxyphenyl)amino)ethylidene)chroman-2,4-dione-palladium(II))complex* (C1). Yellow powder. Yield: 0.048 g (45%). *Anla. calc.* For C_34_H_24_O_8_N_2_Pd (Mr = 694.53) %: C, 58.79; H, 3.48; N, 4.03. Found: C, 58.34; H, 3.75; N, 3.80. IR (KBr) *ν* cm^−1^: 3415 (OH); 3048 (=CH); 2939 (CH); 1681 (C=O); 1602 (C=N); 1560, 1479, and 1428 (C=C); 1161 (C–O); 526 (Pd–O); 457 (Pd–N). ^1^H NMR (200 MHz, DMSO-d_6_) *δ* ppm: 2.59 (s, 3H, C–2′), 6.78 (m, 1H, C–6^″^), 6.80 (m, 1H, C–4^″^), 6.82 (m, 1H, C–5^″^), 7.10 (m, 1H, C–6), 7.23 (m, 1H, C–7), 7.3 (s, 1H, C–2^″^), 7.59 (m, 1H, C–5), 7.99 (dd, 1H, ^3^JH_8_, H_7_ = 7.8 Hz, ^4^JH_8_, H_6_ = 3.2 Hz, C–8–H), 9.98 (bs, 1H, OH), 9.81 (bs, 1H, OH). ^13^C NMR (50 MHz, DMSO-d_6_) *δ* ppm: 24.0 (C–2′), 105.1 (C–3), 111.9 (C–2^″^), 113.9 (C–4^″^), 115.5 (C–5^″^), 115.7 (C–8), 117.4 (C–5), 123.5 (C–6^″^), 126.4 (C–6), 130.2 (C–10), 133.9 (C–7), 147.7 (C–1^″^), 152.3 (C–3^″^), 158.4 (C–9), 161.5 (C–2), 171.9 (C–4), 180.5 (C–1′).

*Bis(3-(1-((4-hydroxyphenyl)amino)ethylidene)chroman-2,4-dione-palladium(II) complex)* (C2). Yellow powder. Yield: 0.049 g (46%). *Anla. calc.* For C_34_H_24_O_8_N_2_Pd (Mr = 694.53) %: C, 58.79; H, 3.48; N, 4.03. Found: C, 59.00; H, 3.51; N, 4.03. IR (KBr) *ν* cm^−1^: 3345 (OH); 3028 (=CH); 2938 (CH); 1670 (C=O); 1602 (C=N); 1547, 1508, 1477, and 1487 (C=C); 1199 (C-O); 527 (Pd-O); 462 (Pd–N). ^1^H NMR (200 MHz, DMSO-d_6_) *δ* ppm: 2.58 (s, 3H, C–2′), 6.71 (m, 2H, C–H–2^″^, C–H–6^″^), 6.87 (m, 2H, C–3^″^–H, C–5^″^–H), 7.07 (m, 1H, C–6–H), 7.25 (m, 1H, C–7–H), 7.63 (m, 1H, C–5–H), 7.99 (dd, 1H, ^3^J H_8_, H_7_ = 12 Hz, ^4^J H_8_, H_6_ = 2.1 Hz, C–8–H), 9.65 (bs, 1H, OH), 9.90 (bs, 1H, OH). ^13^C NMR (50 MHz, DMSO-d_6_) *δ* ppm: 24.3 (C–2′), 105.1 (C–3), 115.8 (C–3^″^, C–5^″^), 116.5 (C–8), 117.5 (C–5), 123.3 (C–2^″^, C–6^″^), 126.3 (C–6), 126.9 (C–10), 127.1 (C–1^″^), 134.5 (C–7), 152.3 (C–4^″^), 157.4 (C–9), 161.2 (C–2), 169.1 (C–4), 180.3 (C–1′).

### 2.4. DFT Calculations

Theoretical calculations of all the investigated compounds were carried out using Gaussian Program Package [29]. All geometric optimizations were done with M06 hybrid meta functional [30] in conjunction with 6-311 + G(d,p) [31] basis set for C, N, O, and H and def2-TZVPD, triple-zeta-valence, and basis set for palladium(II) [32]. The second basis set, def2-TZVPD, contained polarization and diffuse functions, as well as effective core potential. To determine the most stable structure of the ligands, all rotamers of the investigated compounds were examined. The most stable structures were fully reoptimized with no imaginary frequencies present. To evaluate the solvent effect of DMSO, an SMD solvation model was used [33]. For the simulation of the ^13^C and ^1^H NMR spectra of the investigated compounds, GIAO (gauge independent atomic orbital) approach was used [34, 35]. The chemical shifts for all compounds were calculated relative to TMS. The calculated values for all the hydrogen and carbon atoms were subtracted from the corresponding values for carbon and hydrogen atoms of TMS (178.972 and 32.075, respectively).

The structures of the ligands and complexes were determined by using the M06 DFT method. The most stable structures of all investigated compounds were used for the prediction of the NMR spectra. To verify the proposed structures of complexes, experimentally obtained chemical shifts were compared with theoretical ones. For the estimation of the chemical shifts, the GIAO method was used. This method has been shown to give good results for similar chemical compounds [25, 26]. Theoretical chemical shifts, in DMSO and CDCl_3_ as solvents, were calculated relative to TMS. The correlation coefficient (*R*) and AAE values were calculated.

### 2.5. EPR

DEPMPO spin-trap was purchased from Enzo Life Sciences and purified according to the protocol described by Jackson and others [36]. The purified DEPMPO spin-trap was tested and proved by trapping free radicals from standard generator reactions. Hydroxyl radical (^•^OH) was generated using the standard Fenton reaction (Fe^2+^ + H_2_O_2_ → Fe^3+^ + OH^–^ + ^•^OH, 2 mM H_2_O_2_, and 0.66 mM FeSO_4_), in the presence of 0.1 M DEPMPO. Superoxide thermal source SOTS-1 di(4-carboxybenzyl)hyponitrite) (1 mM) was used for the production of superoxide radicals. SOTS-1 was dissolved in 100 mM phosphate buffer, pH 7. Decomposition of SOTS-1 was induced by heating at 37°C for 5 minutes. Prospective iron-mediated production of hydroxyl radical was prevented by iron-chelator deferoxamine-mesylate (1 mM). The examined compounds were dissolved in DMSO and diluted in deionized water of resistivity not less than 18.2 M*Ω*. All compounds were applied in the concentration range between 1 and 50 *μ*M. The final concentration of 5 *μ*M was chosen as appropriate for molecules with antioxidative activity. Liquid samples were placed in a 4 mm quartz capillary which was fitted with a resonator cavity. Spectra were recorded at 293 K using EMXnano EPR spectrometer (Bruker) operating at X-band (9.452 GHz) with the following settings: scan range 200 G; modulation amplitude, 2 G; modulation frequency, 100 kHz; microwave power, 10 mW; time constant, 64 ms; and scanning time, 1 min. All samples were incubated for 3 min before recording. All the measurements were done with three repetitions. Absolute signal intensity, expressed in arbitrary units (mean value ± standard error), was given as a height of the central signal of DEPMPO-^•^OH and DEPMPO-^•^OOH adducts.

### 2.6. ABTS Assay

Compounds AHC, L1, L2, C1, and C2 (5 *μ*M and 50 *μ*M) were tested for antioxidative potential towards ABTS (2,2′-Azino-bis(3-ethylbenzothiazoline-6-sulfonic acid) radical according to the procedure of Arnao et al. [37] modified for microtiter plate. Reaction mixture (0.3 ml) contained 50 mM K-phosphate buffer, 2 mM ABTS, 15 *μ*M hydrogen peroxide, 0.25 *μ*M horseradish peroxidase, and 5 *μ*l ascorbic acid standard (0.05-1 mM) or tested compounds. The results obtained were expressed as *μ*M ascorbic acid equivalents (AAE).

### 2.7. Cell Culture

Cell lines, MRC-5 (ATCC, CCL-171), HCT 166 (ATCC, CCL-247), A375 (ATCC, CRL-1619), and MIA PaCa-2 (ATCC, CRL-1420), were maintained in Dulbecco's Modified Eagle's medium (DMEM), supplemented with 4500 mg/l glucose and 10% fetal bovine serum (FBS) (all from Invitrogen TM, NY, USA) at 37°C and in 5% CO_2_.

### 2.8. Cell Viability Assay

Cells viability was tested using MTS Cell Proliferation Assay (Promega CellTiter 96® Aqueous One Solution Cell Proliferation Assay). Cells (1 × 10^4^/per well) were seeded in 96-well plates a day before treatment and then treated with various concentrations of coumarin or its derivatives (0.1, 0.5, 1, 5, 10, and 50 *μ*M), for 24 hours. After 24 h, the effect of these treatments was monitored on cell's viability using MTS Cell Proliferation Assay. The colorimetric quantification was done using a plate reader (Plate Reader Infinite 200 pro, Tecan).

### 2.9. Apoptosis Assay

MIA PaCa-2 cells were treated with DMSO or 5 *μ*M of complexes C1 and C2. After 24 h, cells were collected from both media (detached cells) and dish surface (attached cells) resuspended in 1x Annexin binding buffer at a final number of 1 × 10^6^ cells/ml. Then, 5 *μ*l of Annexin V (Annexin V, Alexa Fluor1 488 conjugate, Invitrogen™) and 5 *μ*l of propidium iodide (PI-Invitrogen) were added. The cells were gently mixed, incubated for 15 minutes in the dark at RT, and examined by flow cytometer (CyFlow space, Partec). The flow cytometer collected 100.000 events, which were analyzed using the FloMax software for cytometry.

### 2.10. Toxicity against Zebrafish Embryos

For zebrafish embryotoxicity assessments, the general rules of the OECD Guidelines, for the Testing of Chemicals, were followed. All experiments involving zebrafish were performed in compliance with the European directive 86/609/EEC and the ethical guidelines of the Guide for Care and Use of Laboratory Animals of the Institute of Molecular Genetics and Genetic Engineering, University of Belgrade.

Adult zebrafish (*Danio rerio*, wild type) was obtained from a commercial supplier (Pet Center, Belgrade, Serbia), housed in a light- and temperature-controlled facility at 28°C and standard 14 : 10-hour light-dark photoperiod, and regularly fed twice a day with commercially dry flake food (TetraMin™ flakes; Tetra Melle, Germany) supplemented with Artemia nauplii. Embryos were obtained by natural spawning and raised in the embryo water (0.2 g L −1 of Instant Ocean® Salt in distilled water). Collected eggs at the 4- to 16-cell stage were arrayed in 24-well plates, 10 embryos per well, and incubated with 1 ml of embryo water per well containing various concentrations of the tested compounds at 28°C. Embryos were exposed to five concentrations (1, 5, 10, 50, and 100 *μ*M) of compounds AHC, C1, and C2. Embryo water alone was used as the negative control. Experiments were performed three times using 30 embryos per concentration. Endpoints for toxicity evaluation were recorded at 24, 48, 72, 96, and 120 hpf using an inverted microscope (CKX41; Olympus, Tokyo, Japan). At 120 hpf, embryos were inspected for viability, anesthetized by addition of 0.1% (*w*/*v*) tricaine solution (Sigma-Aldrich, St. Louis, MO), photographed, and killed by freezing at −20°C for ≥24 h.

### 2.11. The Activity of Enzymes of Antioxidative Defense

Cell cultures were treated by adding the appropriate concentration of tested compounds diluted in DMSO, or DMSO as a control, to the culture media. After 24 h of incubation, cells were harvested, rinsed in PBS, and then collected after centrifugation (300 × g for 5 min at room temperature), and 2 × 10^6^ cells were suspended in one ml of a medium containing 0.25 M sucrose, 0.1 M EDTA, and 0.05 M Tris-HCl pH 7.4. After sonication (30 s; 20 kHz; 4°C), cell debris were pelleted (14000 × g for 1 min at 4°C), and the supernatant was used as protein extract. Total protein content was estimated with BSA as a standard protein, using Bradford [38] method modified for microtiter plate reader.

Catalase (CAT) activity was determined polarographically, using Clark-type oxygen electrode (Hansatech Oxygraph), essentially by the method of del Rio et al. [39]. The reaction mixture contained 100 mM potassium phosphate buffer pH 7.5 and 12 mM H_2_O_2_. The reaction was started by the addition of protein extract (10 *μ*l).

Glutathione reductase (GR) was measured spectrophotometrically according to Smith et al. [40]. Reaction mixture contained 0.75 mM DTNB, 0.1 mM NADPH, 0.02 mM GSSG, 1 mM EDTA, and 100 mM potassium phosphate buffer pH 7.5 in 1 ml volume. The reaction was started by the addition of 10 *μ*l of the cell protein extract. Absorbance change was monitored at 412 nm, and the rate of reaction was calculated using *ɛ*_(TNB)_ = 14.15 mM^−1^ cm^−1^.

Glutathione S-transferase (GST) activity was measured essentially by the method proposed by Habig et al. [41]. Reaction mixture contained 100 mM potassium phosphate buffer pH 6.5, 1 mM GSH, 1 mM 1-chloro-2,4-dinitrobenzene (CDNB), and 100 *μ*l protein extract in 1 ml volume. Absorbance change was monitored at 340 nm, and enzyme activity was calculated using extinction coefficient for CDNB of *ɛ*_(CDNB)_ = 9.6 mM^−1^ cm^−1^.

Native PAGE was run on an 8% running gel, according to the method proposed by Laemmli [42]. For each sample, the volume containing 20 *μ*g of protein was applied to the gel. In-gel Superoxide Dismutase (SOD) activity was detected by incubating gels in the dark, in 100 mM K-phosphate buffer (pH 7.8) containing 25 *μ*M NBT and 8.4 *μ*M riboflavin for 30 min [43]. Gels were then thoroughly rinsed in deionized water and exposed to UV irradiation. To distinguish between Mn-, Cu/Zn-, and Fe-SOD, gels were incubated, before activity staining, in the buffer containing 5 mM KCN or 5 mM H_2_O_2_.

### 2.12. Statistical Analyses

The data represent the means ± SEM from at least three independent experiments. Statistical analyses of *in vitro cytotoxicity assay* and a*poptosis assay data* were performed by Student's *t*-test. To take into account both effects of the tested compounds and cell types, the activities of the antioxidant enzymes were compared statistically using a two-way analysis of variance (two-way ANOVA). Pairwise comparisons were made with the Holm-Sidak test. The results were considered to be statistically significant at *P* < 0.05. The Student's *t*-test and two-way ANOVA were performed with the aid of the Sigma Plot 12 software (Systat Software Inc., USA).

Dependence of the cytotoxic effects of the investigated compounds upon their effect on the activity of the antioxidative defense system enzymes, was tested using Partial least square regression (PLSR) (XLSTAT statistical and data analysis solution, Addinsoft 2019, Boston, USA).

### 2.13. A Protocol of Molecular Docking Study

The molecular docking simulations were employed to better understand the inhibitor efficiency of coumarin derivatives, ligands, and their palladium(II) complexes. The binding affinity of the investigated compounds was examined by the AutoDock 4.2 software [44]. For this purpose, the X-ray crystal structure of the protein was used. The crystal structure of the receptor tyrosine kinase protein (PDB: 3GQL) [45] was downloaded from RCSB Protein Data Bank in PDB format. The protein was prepared for docking by removing the cocrystallized ligand, water molecules, and cofactors in Discovery Studio 4.0. [46]. The Kollman partial charges and polar hydrogens were added using the AutoDockTools (ADT) graphical interface [44]. The ligands and their complexes were prepared for docking by minimizing their energy at the previously mentioned DFT level of theory. In ADT, the flexibility of the ligands/complexes was considered, while the protein was kept on as the rigid structure. To express their flexibility, the bonds of ligands/complexes were set to be rotatable. The Lamarckian Genetic Algorithm (LGA) method was used for protein-ligand/complexes flexible docking. The parameters for the LGA method were determined as follows: a maximum number of energy evaluations was 250,000; a maximum number of generations was 27,000; and mutation and crossover rates were 0.02 and 0.8, respectively. The AutoDock 4.2 software was based on algorithms that can predict compound's poses within the protein target and assess them by scoring functions by setting the grid box. The grid boxes with dimensions 58 × 74 × 109 Å^3^ in *-x*, *-y*, and *-z* directions of RTK protein were used to cover the protein binding sites and accommodate ligands to move freely. A gridpoint spacing of 0.375 Å was used for Auto Grid runs. The three-dimensional (3D) results of the interactions between the target protein and the investigated compounds were analyzed and illustrated in Discovery Studio 4.0 and AutoDockTools.

## 3. Results

### 3.1. Chemistry

The synthesis of new derivatives of 4-hydroxycoumarin and their corresponding palladium(II) complexes was carried out hoping to obtain new biologically active compounds. The synthesis of two novel ligands was performed in two steps as presented in Scheme 1. In the first step, 4 hydroxycoumarin was acylated to give 3-acetyl-4-hydroxycoumarin (AHC). In the next step, the obtained compound AHC was coupled with meta- and para aminophenols and gave new compounds, L1 and L2. The complexation reaction was performed by mixing equimolar amounts of ligands and potassium tetrachloropalladate(II) K_2_[PdCl_4_], resulting in the formation of palladium(II) complexes, C1 and C2 (Scheme 1).

### 3.2. Spectroscopic and DFT Characterization

Structural features of the examined compounds were determined by nuclear magnetic resonance (NMR) and infrared (IR) spectroscopy and elemental analysis (see more details in experimental section), as well as DFT methods.

Interpretation of the obtained NMR spectra was facilitated by the use of literature data in which, in addition to the application of NMR spectroscopy, the structure of the compounds, which are structurally similar to the compounds analyzed in this paper, was determined by X-ray crystallography [47–49]. Spectral data obtained by ^1^H NMR revealed/confirmed the aromatic structure of the newly synthesized ligands by the existence of several signals within the characteristic range of the chemical shift values, between 6.77 and 7.95 ppm. Singlets at 2.55 (L1) and 2.57 (L2) ppm can be assigned to protons of the methyl groups at C2′ of both ligands. The presence of OH groups at *m*-hydroxyaniline and *p*-hydroxyaniline appeared as broad singlets, positioned at 9.99 ppm (L1) and 9.91 ppm (L2). As expected, the peak of the proton belonging to the amine functional group appeared as a singlet positioned at the highest frequencies, at 15.42 and 15.28 ppm, respectively. The ^1^H NMR spectra of C1 and C2 complexes were deprived of signals originated from the amino group, which is evidence that, before coordination to palladium(II) ion, the ligands were deprotonated. The chemical shifts of other protons in complexes are similar to the corresponding ones in the ligands' spectra.

In ^13^C NMR spectra, the chemical shifts for C atoms of the coumarin moiety, for both ligands, were in the range of 97.06–175.80 ppm, while the corresponding signals of the aminophenol ring were moved downfield in the interval of 112.5–153.4 ppm. Most download shifts were detected for C1′ because of its bridging position between electronegative nitrogen and coumarin moiety. The signal from the most shielded carbon of the methyl group was detected at 20.5 ppm. Comparison of ^13^C NMR spectra of ligands and complexes revealed differences in the chemical shifts of a C4 carbon atom, which are shifted downfield relative to the corresponding ones in the ligands' spectra. This is expected given the fact that during the coordination of ligands and palladium(II) ion, a new bond is formed between Pd and O, which is followed by the transformation/reduction of the carbonyl group C=O into C–O. Moreover, the transformation of the C3–C4 single bond into a double bond contributed to the greater chemical shift of the C3 atoms in the complexes. Also, as evidence of the formation of a bond between palladium(II) and nitrogen is a chemical shift of C2′. This carbon atom was shifted downfield by 3.6 ppm (C1) and 3.8 ppm (C2).

Analysis of the IR spectra of two ligands confirmed the presence of OH group to which a broad band, in the region 3228–3345 cm^−1^, was assigned. The IR spectra also revealed bands assigned to vibrations of the NH group, in the 3228–3259 cm^−1^ range, and to stretching vibrations of the lactone carbonyl groups in the range of 1675–1687 cm^−1^. As opposed to ligands, IR spectra of the synthesized complexes showed no bands that could be assigned to NH vibrations. In the given spectra, the band positioned at 1602 cm^−1^ was assigned to C=N stretching vibration just like bands at 526 cm^−1^ and 457 cm^−1^ assigned to stretching vibrations of Pd-O and Pd-N bonds.

The theoretical structural characterization of the ligands and complexes was performed by applying the M06 DFT method. The corresponding values of bond lengths and bond angles for all investigated compounds are given in Tables S1 and S2. The optimized structures are presented in Figure S1. Experimental and calculated ^1^H NMR and ^13^C NMR chemical shifts, obtained at DFT/B3LYP-D3BJ level of theory, are presented in Tables S3 and S4. The values of the correlation coefficients (*R*) and relatively small values of AAE (Tables S3 and S4) indicated a good linear correlation between experimentally obtained and calculated values proving the applied theoretical model as adequate.

### 3.3. Antiradical Activity

**Figure 1:**
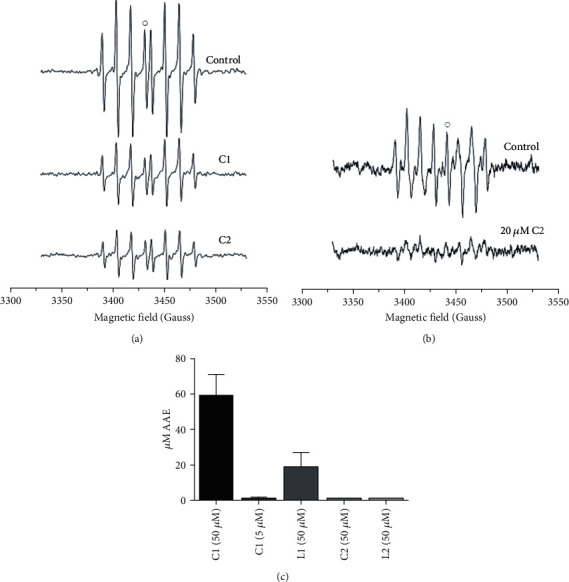
EPR spectra of DEPMPO/OH spin-adduct upon addition of 5 *μ*M C1 and C2 (a) and DEPMPO-^•^OOH adduct upon addition of 20 *μ*M C2 (b). The antioxidant activity is calculated relative to the heights of the peaks marked with open circles; ABTS quenching activity of C1, L1, C2, and L2. Concentrations tested are given in brackets (c).

The antiradical activity of the selected compounds toward ^•^OH radical was examined by EPR. The standard Fenton reaction system was used for the generation of ^•^OH radicals to a high extent.  Figure 1 shows EPR spectra of stable DEPMPO/OH spin-adducts in 0.1% DMSO, before and after the addition of C1 and C2. It is observed that the addition of C1 and C2 to the Fenton reaction system decreased, to an almost equal extent, the amount of DEPMPO/OH adduct. A slightly more pronounced reduction effect, approximately 68% (from 83.51 ± 4.14 a.u to 28.04 ± 0.99 a.u), was shown by complex C2 (5 *μ*M). The reduction percentage of the complex C1 (5*μ*M) was approximately 62% (from 83.51 ± 4.14 a.u to 31.51 ± 0.79 a.u). Unlike C1 and C2, their corresponding ligands L1 and L2 demonstrated no activity towards ^•^OH just like AHC even when applied at a concentration as high as 50 *μ*M.

The antiradical activity towards superoxide radicals, generated by the decomposition of the SOTS-1 as initiator, was also assessed by EPR [50]. No observable changes in the intensity of spectra of DEPMPO-^•^OOH adduct were obtained after the addition of the AHC and C1 complex in the concentration range of 1-50 *μ*M. However, the addition of 20 *μ*M C2 complex induced reduction of the DEPMPO-^•^OOH adduct by approximately 78% (from 179.3 ± 4.9 a.u to 38.4 ± 1.3 a.u, Figure 1).

The effect on 2,2′-Azino-bis(3-ethylbenzothiazoline-6-sulfonic acid) cation radical (ABTS) quenching was noticed after the addition of 50 *μ*M C1 complex or its corresponding ligand, L1. C1 complex showed antioxidative activity of 59 ± 12 *μ*M AAE, while the activity of L1 was significantly lower, 19 ± 8 *μ*M AAE, *P* = 0.049. At lower concentrations, complex C1 (5 *μ*M) has shown low and statistically insignificant antiradical potential (Figure 1(c)). The antiradical activity of all other compounds tested, AHC, L2, and C2, was below the detection limit.

### 3.4. In Vitro Cytotoxicity

**Figure 2:**
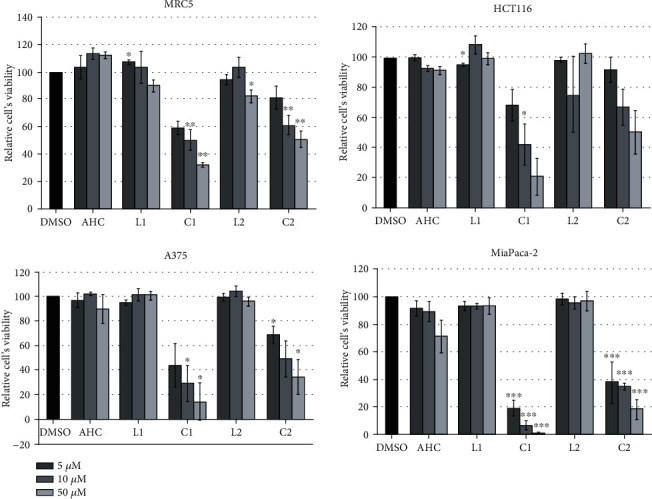
In vitro cytotoxicity assay. Healthy fibroblasts (MRC-5) and three different carcinoma cell lines (HCT 116, A375, and MIA PaCa-2) were exposed to DMSO as a negative control, AHC as a starting coumarin compound, its derivates L1 and L2, and the corresponding palladium(II) complexes C1 and C2. Relative cells' viability was calculated as a percentage of DMSO treated cell viability that was set as 100%. Data are presented as the means ± S.E.M. (standard error mean) of at least three independent experiments performed in 3-plicates for each concentration. Mean values of relative cell viability were compared with Student's *t*-test, and *P* values are presented as ^∗^*P* ≤ 0.05, ^∗∗^*P* ≤ 0.01, and ^∗∗∗^*P* ≤ 0.001. Each color corresponds to a bar presented on the histogram.

To test the cytotoxic potential of newly synthesized ligands and complexes, four different cell lines, colorectal carcinoma (HCT166), melanoma (A375), pancreatic carcinoma (MIA PaCa-2), and one healthy fibroblast cell line (MRC-5) were subjected to treatments with either DMSO, as a negative control, AHC as a starting compound, derivatives L1 and L2, or the corresponding palladium(II) complexes C1 and C2. Five different concentrations were used ranging from 0.1 *μ*M to 50 *μ*M. The viability of cells was tested by MTS assay 24 h upon treatments. Low concentrations, 0.1, 0.5, and 1 *μ*M, of all tested compounds, exhibited no cytotoxic effect on any cell line (data not shown). However, concentrations above 5 *μ*M (5, 10, and 50 *μ*M were tested) of complexes C1 and C2 showed a significant cytotoxic effect with the most prominent effect on the MIA PaCa-2 cell line (Figure 2). The second in line in sensitivity was melanoma cell line A375, while HCT116 cells appeared to be least sensitive. In particular, even 5 *μ*M of C1 led to an overall 80% reduction in MIA PaCa-2 cells' viability after 24 h. With higher concentrations, reduction was above 90%. Calculated IC_50_ values for tested C1 complex were 3 *μ*M for MIA Paca-2 cell line, 5.4 *μ*M for A375 cell line, 7 *μ*M for HCT116 cell line, and 8.8 *μ*M for MRC-5 cells. Regarding the effect of the second complex, C2, it was approximately 2.5 times less effective compared to C1, with IC_50_ values: 6 *μ*M for MIA Paca-2 cell line, 17 *μ*M for A375 cell line, 14.5 *μ*M for HCT116 cell line, and 25 *μ*M for MRC-5 cells (Table 1). It is important to point out that a healthy fibroblast cell line (MRC-5) was the least sensitive to treatments. Although a significant effect was observed in A375 and HCT116 cells as well, the high sensitivity of pancreatic carcinoma cell line indicated some cell-line specificity.

### 3.5. Apoptosis Assay

**Figure 3:**
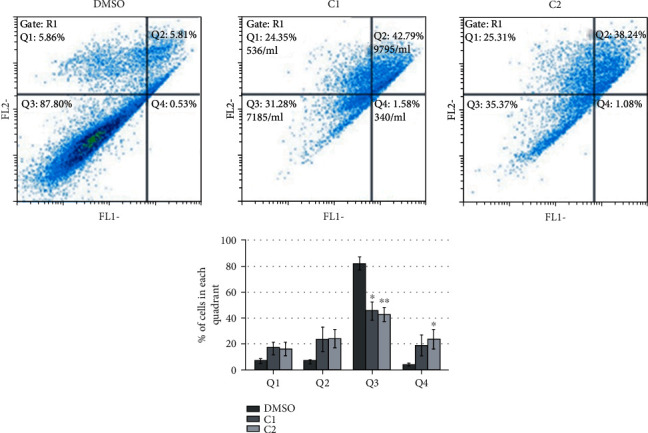
Apoptosis assay. MIA PaCa-2 cells were treated with DMSO as a negative control and coumarin complexes C1 and C2. 24 h upon treatment, cells were collected, and Annexin V-FITC assay was performed. The percentage of cells in each quadrant is presented as the means ± S.E.M. (standard error mean) of at least three independent experiments. Mean values were compared with Student's *t*-test, and *P* values that were evaluated to DMSO treatment are presented as ^∗^*P* ≤ 0.05, ^∗∗^*P* ≤ 0.01, and ^∗∗∗^*P* ≤ 0.001. Each color corresponds to a bar presented on the histogram. Q1: necrotic cells; Q2: late apoptosis; Q3: living cells; Q4: early apoptosis.

Since coumarin complexes C1 and C2 exhibited the highest cytotoxic effect, particularly against MIA PaCa-2 cells, given compounds were further tested to determine the type of cell death they caused on this particular cell line. In previously used cytotoxicity assays, we used concentrations ranging from 0.1 *μ*M to 50 *μ*M. In this study, a concentration of 5 *μ*M was used, which in previous experiments led to a significant reduction in viability. Cells were treated with 5 *μ*M of C1 and C2 and after 24 h collected for Annexin V-FITC assay. As presented in Figure 3, there is an induction of cell death upon treatment of cells with either C1 or C2 compared to DMSO treatment. Upon treatment with C1 or C2, around 45-50% of the cells were in the early or late apoptotic phase (Q2) and approximately 20% in the necrotic phase (Q1) compared to about 10% of cells in both phases upon DMSO treatment. Therefore, it could be stated that detected reduction in cells' viability is due to induction of cell death by tested complexes.

### 3.6. Toxicity Testing on Zebrafish Embryos

**Figure 4:**
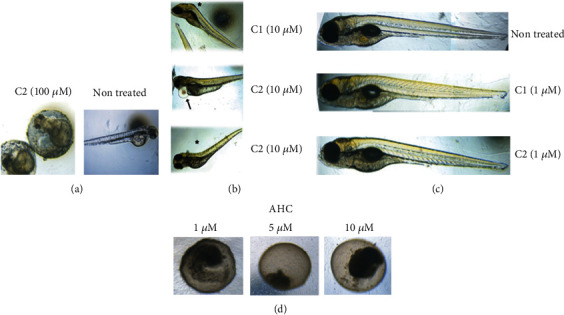
Zebrafish toxicity testing. (a) Representative image of unsuccessful hatching upon treatment of zebrafish embryos with 100 *μ*M of coumarin complex C2 (left panel) compared to successful hatching of nontreated embryos (right panel). (b) Skeletal (asterisk) and cardiovascular abnormalities (arrow) detected at 10 *μ*M concentration of either C1 or C2. (c) Representative images of normal development of nontreated embryos or treated with 1 *μ*M of C1 or C2. (d) Lethal effect of starting coumarin compound (AHC) detected within first 24 h, at any tested concentration.

The effects of AHC and two coumarin complexes, C1 and C2 that exhibited cytotoxic effects *in vitro*, were also examined *in vivo* by monitoring the development of the *Danio rerio* (zebrafish) model system. The zebrafish model, which develops many cancer types of comparable signaling pathways and morphology as in humans, allowed high-quality *in vivo* toxicological testing of new coumarin derivatives. A range of different concentrations has been used (1 *μ*M, 5 *μ*M, 10 *μ*M, 50 *μ*M, and 100 *μ*M), and the effect monitored up to 120 hpf (hours postfertilization). At 50 and 100 *μ*M concentrations of C1 or C2, the majority of treated embryos were prevented from hatching (Figure 4(a), left panel) compared to nontreated embryos that were successfully hatched (Figure 4(a), right panel). Hatching success is a sensitive endpoint of zebrafish development since no hatched embryos could be considered as a lethal outcome. Upon treatment with 10 *μ*M of C1 or C2, hatching was completed but with the appearance of several adverse developmental effects including skeletal and cardiovascular abnormalities, as presented in Figure 4(b). At a concentration of 5 *μ*M, the majority of the embryos exhibited normal development with few showing growth delay or, rarely, skeletal abnormalities (data not shown). Finally, at 1 *μ*M of either C1 or C2, no increase in adverse developmental effects was detected compared to nontreated embryos (Figure 4(c)). Importantly, unlike C1 and C2, where the drop in concentration resulted in a reduction of embryotoxicity, AHC showed a pronounced embryotoxic effect that caused necrotic events even at 1 *μ*M concentration (Figure 4(d)).

### 3.7. Effects on the Activity of the Antioxidative Defense (AOD) System Enzymes

In pursuit of the potential mechanism of cytotoxic effects of C1 and C2, based on their antiradical activities towards hydroxyl (Figure 1(a)) and superoxide (only C2, Figure 1(b)) radicals, effects of these complexes on the activity of major enzymes of the AOD system, namely, GST, GR, CAT, MnSOD, and CuZnSOD, were investigated. Enzyme activities were measured in a healthy fibroblast cell line (MRC-5) as control and the most affected carcinoma cell line, MIA PaCa-2.

**Figure 5:**
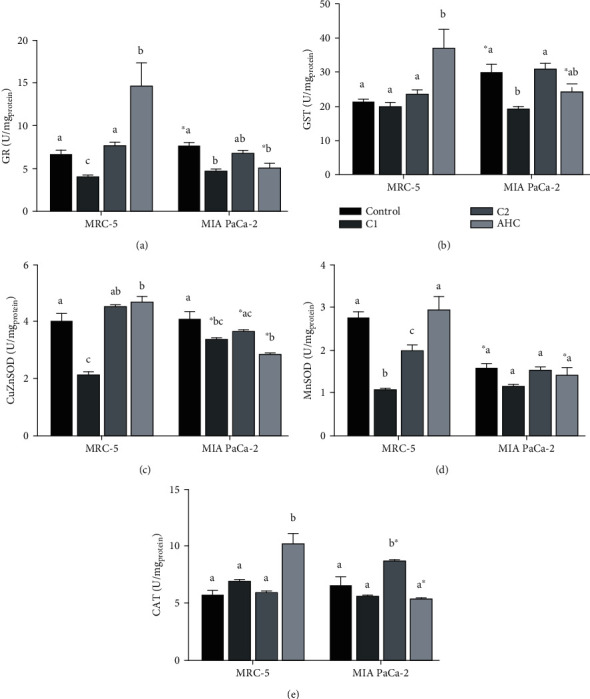
Effect of the selected compounds (AHC and complexes C1 and C2) on the activity of enzymes of the antioxidative defense system: GR (a), GST (b), CuZnSOD (c), MnSOD (d), and CAT (e) in a healthy fibroblast cell line (MRC-5) and carcinoma cell line (MIA PaCa-2). Effects of the selected compounds on the same cell line that are significantly different (*P* < 0.05) are marked with different letters (a, b, c). A statistically significant difference (*P* < 0.05) between effects of the same treatment on MRC-5 and MIA PaCa-2 cell lines is marked with an asterisk (^∗^).

The lowest concentrations of C1 and C2 complexes (5 *μ*M, Figure 2) with significant cytotoxic effect on MIA PaCa-2 were applied. The effect of 50 *μ*M AHC, as a parent compound, on the activities of enzymes was also measured since this concentration showed a small stimulatory effect on healthy cell growth and the most adverse cytotoxic effect on MIA PaCa-2 cells (Figures 2(a) and 2(d)). As in the cytotoxicity test, the activities of enzymes were measured after 24 h, and DMSO-treated cells were used as a control. Except for MnSOD and GST, there were no statistically significant differences in the activity of enzymes between MIA PaCa-2 and MRC-5 cells in the control treatment (Figure 5). However, each of the compounds used showed distinctive effects on the activity of enzymes (Figure 5). In healthy cells, AHC induced a statistically significant increase in the activity of all enzymes except MnSOD. However, when tested on the MIA PaCa-2 cell line, it had either no effect (GST, MnSOD, CAT, Figures 5(a), 5(d), and 5(e)) or induced a statistically significant decrease (GR, CuZnSOD, Figures 5(b) and 5(c)). C1 reduced the activity of most enzymes except CAT and GST in healthy cells and CAT and MnSOD in the MIA PaCa-2 cell line (Figure 5). It is important to emphasize that the activity of C1 was partially selective since it reduced the activity of MnSOD only in healthy cells (Figure 5(d)) and the activity of GST only in cancer cells (Figure 5(a)). The C2 complex mainly induced no effects, except for decreasing MnSOD activity in healthy cells (Figure 5(d)) and increasing CAT activity in a carcinoma cell line (Figure 5(e)), which was the only statistically significant effect on CAT activity in carcinoma cells.

**Figure 6:**
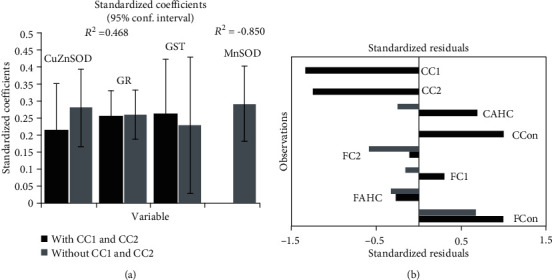
Quality of the Partial least square regression (PLSR) model describing the dependence of the cytotoxic effects of the investigated compounds (AHC and complexes C1 and C2) on the activity of the antioxidative defense system enzymes (GST, GR, MnSOD, CuZnSOD, with (black bars) and without (grey bars) CC1 and CC2 observations) present in a healthy fibroblast cell line (FAHC, FC1, and FC2, respectively) and pancreatic carcinoma cell line (CAHC, CC1, and CC2, respectively) (a). PLSR standardized regression coefficients. The bars indicate 95% confidence intervals based on jackknifing. Bar chart of the standardized residuals (b).

Partial least square regression (PLSR) was used to test the dependence of the cytotoxic effects (dependent variable, *Y*, Figure 6) of the investigated compounds (AHC and complexes C1 and C2) on the activity of the antioxidative defense system enzymes (independent variables, *X*, Figure 6) present in healthy fibroblast cell line (MRC-5) and pancreatic carcinoma cell line (MIA PaCa-2). To obtain a model based on the combination of enzyme activities with statistically significant influence on the measured cytotoxic effect, the model was pruned until all variables having insignificant standardized regression coefficients (confidence intervals include 0), in this case CAT and MnSOD, were deleted. Bar graphs of the statistically significant standardized regression coefficients for the model are shown in Figure 6(a). Upward pointing bars indicate a positive influence on cytotoxicity. However, the *R*^2^ value of the model was only 0.468, meaning that at the best 46.8% of the variability in cytotoxic effects of the investigated compounds can be explained by their coordinated action on the activity of GST, GR, and CuZnSOD. Analysis of model residuals showed that the largest residuals characterize effects of C1 and C2 on carcinoma cell line (CC1 and CC2, respectively, Figure 6(b)), while the smallest ones characterize effects of these compounds on healthy fibroblast cell line (FC1 and FC2, respectively). This flagged CC1 and CC2 as potential outliers. Since such deviation of CC1 and CC2 from other samples could potentially mask any systematic trends among the other samples, the PLS was repeated with CC1 and CC2 excluded. It resulted in a model with much greater explanatory power (*R*^2^ = 0.850), which additionally included the effect on MnSOD activity (Figure 6(a)). It can be seen that residuals in this model are much smaller (Figure 6(b)). Obtained results indicate that the majority of the overall cytotoxic effect of the compounds tested has come from the interactions with antioxidative enzyme system, except the effect of C1 and C2 on cancer cells, which has some additional or synergistic component.

### 3.8. Molecular Docking Study

To evaluate the inhibitory activities of ligands/complexes against some proteins, a docking analysis is performed for different groups of molecules, showing the most significant correlation with receptor tyrosine kinase, RTK. In Table S5 and Figure S2, the most stable docking conformations of ligands and their corresponding metal complexes are presented. The lower value of K_i_ and the more negative value of Δ*G*_bind_ indicated better inhibition. The values presented in Table S6 indicate strong binding of L1 and L2, and their metal complexes C1 and C2, to receptor tyrosine kinase. The docking analyses of the investigated molecules revealed the existence of several noncovalent interactions between the target molecules and RTK. The most prominent interactions are hydrogen bonds, alkyl-*π*, and *π*-*π* interactions (Table S5).

## 4. Discussion

In this paper, the synthesis of novel coumarin derivatives is presented. Starting from the weak antioxidant, AHC molecule, in coupling reactions with different aminophenols, two new ligands, and their palladium(II) complexes were obtained. Although the new ligands did not show better antioxidant or cytotoxic effects than the parent molecule, AHC, their palladium(II) complexes did.

Presented results on antiradical activity of C1 and C2 are in agreement with the literature data, related to the activity naturally occurring and synthesized coumarin-based compounds, by which the activity of the same scaffold, among others, varies with the number of hydroxyl groups [51]. EPR experiments proved the selective antioxidant activity of C1 and C2. While C1 showed activity towards ^•^OH and ABTS radicals and no activity towards ^•^OOH, C2 complex showed more pronounced activity towards both ^•^OH and ^•^OOH radicals and no ability to reduce ABTS. The reduction ability of C1 towards ABTS was comparable to that of ascorbic acid. The presented result suggested that C1 and C2 display significant radical scavenging effects on biologically relevant ^•^OH and ^•^OOH radicals that may help prevent or ameliorate oxidative damage. It is possible to assume the influence of palladium ion on the antiradical activity as a contributing factor. The exact and relevant reaction mechanism of their reduction abilities is in progress and will be published in the future as a detailed SAR study.

*In vitro* results on cytotoxic effects of new compounds clearly demonstrated the potential of C1 and C2 to significantly reduce cells' viability, especially against pancreatic carcinoma MIA PaCa-2 cell line, mostly by inducing apoptosis (Figure 3). These results are very significant taking in mind the fact that pancreatic cancer is highly chemo-resistant, which in recent years has initiated extensive research into new drugs. The results indicate above 90% of the reduction in MIA PaCa-2 cells' viability when cells were treated with 50 *μ*M C1. Even at ten times lower concentrations, the cytotoxic effect was considerable and proved by the reduction in cells' viability up to 80% (C1) and 60% (C2). Obtained complexes are proved very potent in terms of their antiproliferative effect that is even more important having in mind that the same derivatives have shown significantly lower cytotoxic potential on fibroblast deriving from normal tissue (MRC-5 cells). The difference in response between carcinoma and healthy cells could be a base for further investigation and improvement of these compounds. The results are also in agreement with the literature data related to some coumarin-containing compounds showing specific antitumor activity against pancreatic carcinoma [52, 53].

*In vivo* experiments performed on the zebrafish model have shown the embryotoxicity of the complexes ranging from low to complete absence of toxicity, at concentrations of 5 *μ*M and less. Further testing will be necessary to determine the optimal effective concentration that will induce specific anticancer activity but not embryotoxicity.

Increased ROS load is a hallmark of any type of cancer [54, 55], and consequently, the effectiveness of antioxidative defense is important in cancer. Generally, overexpression of the MnSOD leads to the inhibition of growth rate and the invasiveness of some types of cancer. Compared to normal cells, the level of MnSOD in some cancer-type cells is elevated contributing to the aggressiveness of cancer which is directly related to the elevated enzyme level. However, pancreatic cancer cell lines, as well as cells obtained from pancreatic tumor patients, have been found to have the faulty signaling pathway of activation of the major transcription factor that orchestrates antioxidative response (SIRT3-NRF2-ARE pathway), with a decreased level of SIRT3, paradoxically highly activated NRF2, but the very low activity of MnSOD [56–60]. Following the literature cited here, presented results also indicate a significantly lower level of MnSOD in MiaPaCa-2 cells compared to healthy fibroblast controls. As it is expected, the applied PLSR model indicates that under exposure to the compounds tested, the cell viability, in all groups, is positively correlated with the increased activity of enzymes of the antioxidative defense. For instance, AHC increased enzyme activity and promoted the viability of healthy fibroblast cells. Not fitting in the PLSR model were experimental groups of C1 and C2 acting on pancreatic cancer cells, suggesting that interaction of C1 and C2 with MiaPaca-2 cells is somehow specific and different from the effect the same compounds have on healthy fibroblasts.

As in the cytotoxicity assay, the effects of complexes on antioxidant defense pathway enzymes (AOD) deviated significantly from the effects of the parent compound AHC, demonstrating that complexes have new, different, properties. AHC showed interesting effects indicating that in cells, it acts like a prooxidant, with SOD-inducing activity. Increased CAT activity in healthy cells was again induced only by AHC, possibly a consequence of the elevated peroxide production.

Recently, data have been published indicating that the increased activation of AOD actually confers cisplatin resistance in lung cancer cell lines [61]. On the other hand, the negative side effects of cisplatin, unwanted cytotoxicity of healthy cells, are accompanied by a decrease in antioxidative capacity as measured by a decrease in AOD enzymes [62–64]. While increased AOD enzyme activity in response to ROS is a physiological process tightly regulated by an NRF2 pathway, increased NRF2 activation in cancer cells can lead to adaptation to stress and resistance to treatment [65]. On the other hand, the decline in AOD enzyme activity is interpreted as a loss of antioxidative capacity, possibly as a consequence of the deactivation of enzymes by excess ROS [66]. Ideally, a good novel compound with selective anticancer activity would decrease AOD enzymes minimally or not at all in healthy cells, while avoiding increased activation of AOD in the course of prolonged treatment in malignant cells. Currently, it is proposed that a balance between causation of ROS-mediated damage (cytotoxicity) and avoidance of strong activation of NRF2 pathway (adaptation leading to chemoresistance) is attainable by combined application of various antioxidants and established anticancer drugs [67]. The Pd(II)-coumarin complexes presented in this manuscript have promising effects in that same direction.

We have shown that at C1 and C2 concentration cytotoxic for cancer cells, enzymes of AOD are mostly spared or somewhat downregulated, in both cell lines treated. Notable is the finding that C1 induces the reduction of major detoxifying enzyme GST in the pancreatic cancer cell line, potentially making those cells more vulnerable to further treatment [68]. In contrast, C2 had no activity towards glutathione-related enzymes GST and GR, while strongly inducing CAT. The usual cause of CAT increase is elevated peroxide production suggesting that, inside the cell, C2 somehow promoted peroxide generation. It is possible to assume that C2 gets biotransformed into a CAT-inducing molecule, or goes, similar to ascorbate in high concentrations, through prooxidative cycling producing hydrogen peroxide [69]. Alternatively, C2 could also interact with component(s) of a signaling pathway leading to alternations in CAT and SOD activities, as reported for 7-hydroxy-3-(4′-methoxyphenyl) coumarin [70].

The C1 and C2 have similar, albeit not identical impacts on MRC5 and MIA PaCa-2 SOD enzymes. Notable reduction of both Mn-SOD and Cu-Zn SOD in both cell lines suggests that antioxidative capacity for superoxide radical is dampened by C1 and up to some extent by C2, with C1 having a more prominent unwanted effect in healthy control cells. In contrast to C1, C2 is active towards superoxide radical (as shown by EPR measurements), and therefore, it is possible to assume that C2 mitigates some negative effects through its antiradical activity. The AOD effects of C1, a more potent apoptosis inducer, were strikingly different than C2, lowering glutathione–related enzymes in cancer cells. Since in healthy cells, GR activity decreased as well, and C1 exhibited a cytotoxic effect on healthy cells (although smaller than on MIA PaCa-2), that is probably the effect that in future studies should be explored and mitigated. It is important to emphasize that the interaction with AOD enzymes is not the primary apoptosis-inducing mechanism in cancer cells, as demonstrated by the PLSR model, which indicates that the mechanism of cancer cells killing by C1 and C2 needs to be explored further. The specifically large effect on cancer lines with known NRF2 activated signaling and consistent reduction of MnSOD activity indicates that C1 and C2 might interact with some component of the SIRT3-NRF2 pathway. Recently published data on 7-hydroxy-3-(4′-methoxyphenyl) coumarin report on its affinity for SIRT3 and the ability to activate it and subsequently increase SOD2 activity [70]. Based on the results obtained, it is possible to assume that biological effects of C1 and C2 are partially mediated via inhibitory interaction with molecules in the Sirt3-NRF2 pathway, bringing MnSOD activity even lower and orchestrating the collapse of the antioxidative defense in cancer cells.

Following known facts that the excess ROS stimulate tyrosine kinases to activate NRF2 and that in many carcinomas, including pancreatic carcinoma, there is an overexpression of various tyrosine kinase receptors and their ligands, docking studies identified family of tyrosine kinase receptors as a possible important drug target for tested coumarin derivatives and their corresponding palladium(II) complexes. Tyrosine kinase inhibitors target specific tumor pathways associated with carcinogenesis. Although the inhibition of tyrosine kinases has been viewed as a promising approach for the treatment of pancreatic cancer preliminary results of trials with tyrosine kinase inhibitors have been disappointing regarding the treatment of this specific cancer [71]. There have also been many reports on various coumarin-containing compounds entering possible interaction with tyrosine kinase receptors in different carcinomas, for example, breast carcinoma that expresses EGFR [72].

**Figure 7:**
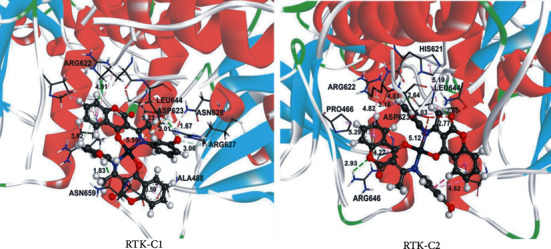
The hydrogen bond (green dotted lines) and hydrophobic (rose pink dotted lines) docking interactions of the most stable conformation of C1 and C2 with RTK protein.

According to docking studies, coumarin complexes C1 and C2, exhibiting *in vitro* potent antiproliferative effect against pancreatic carcinoma cells, can interact with tyrosine kinase receptors. Docking analysis implies that C1 and C2 bind RTK more effectively in comparison to their corresponding ligands (Figures 7 and S2 and Table S6), which is also following the experimental results on cytotoxicity activity (Figure 2). The results indicate that C1 has more negative binding energy and a lower value of inhibition constant (Δ*G*_bind_ = −44.7 kJ/mol, *K*_*i*_ = 0.01 *μ*M). C1 enters significant interactions with RTK establishing three hydrogen bonds *via* the amino acid residues ASP641, ASN659, and ARG646, one alkyl-alkyl, and four *π*-alkyl interactions (Table S5). Both C1 and C2 have lower binding free energy (Δ*G*_bind_) values and consequently show better inhibitory activity in comparison to other palladium(II) complexes [73–77]. Further investigation is needed to completely understand the mechanism of action of tested complexes and explore their prospective in carcinoma treatments.

## 5. Conclusion

In this study, two new 4-hydroxycoumarin bidentate ligands and their palladium(II) complexes have been successively synthesized, structurally characterized, and screened for biological activity. Obtained compounds showed selective antioxidant activity towards ^•^OH, ^•^OOH, and ABTS radicals. *In vitro* cytotoxicity, tested on three different carcinoma cell lines (HCT166, A375, and MIA Paca-2) and one healthy fibroblast cell line (MRC5), was cell-type selective and dependent upon the concentration of the tested complexes. The mode of cell death induced upon the action of complexes pointed to the early or late apoptotic and necrotic phases. *In vivo* cytotoxicity tested on zebrafish embryos, dependent upon the concentration of AHC and tested complexes, varied from preventing hatching to several developmental effects including skeletal and cardiovascular abnormalities. Investigated complexes showed different effects on AOD enzymes while the docking analysis proved the possible interaction of complexes with tyrosine kinase receptors and better inhibitory activity of C1 in comparison with other palladium(II) anticancer complexes.

In conclusion, preliminary studies imply the effect of C1 and C2 on pancreatic carcinoma cells as specific and more toxic compared to the effect on healthy cells. These types of compounds could be a useful starting point in future drug development.

## Figures and Tables

**Table 1:** IC_50_ values for complexes C1 and C2.

Cell line	IC_50_ (*μ*M)	
	C1	C2
MIA Paca-2	3.0	6.0
A375	5.4	17.0
HCT116	7.0	14.5
MRC-5	8.8	25.0

## Data Availability

The supplementary information contains additional information on experimental and calculated ^1^H and ^13^C NMR chemical shifts, bond lengths and angles of newly synthesized molecules, as well as results of docking analysis for the most stable conformations of complexes C1 and C2 with RTK protein (type of interaction, atom distances, binding free energies, and inhibition constants).

## Supplementary Materials

Supplementary Materials Table S1: bond lengths of the investigation compounds: L1, L2, C1, and C2. Table S2: bond angles of the investigation compounds: L1, L2, C1, and C2. Table S3: experimental and calculated (at DFT/B3LYP-D3BJ level of theory) ^1^H NMR chemical shifts (ppm, in DMSO) of the investigation compounds. Table S4: experimental and calculated (at DFT/B3LYP-D3BJ level of theory) ^13^C NMR chemical shifts (ppm, in DMSO) of the investigation compounds. Table S5: important interactions between amino acids of RTK receptor and the investigated compounds, estimated hydrogen bond, and important thermodynamic parameters: binding free energy (Δ*G*_bind_) and constant of inhibition (*K*_*i*_). Table S6: the important thermodynamic parameters for best docking conformations of ligands and palladium complexes with RTK protein: RTK-L1, RTK-L2, RTK-C1, and RTK-C2. Figure S1: optimized structures of ligands and corresponding complexes. Figure S2: the hydrogen bond (green dotted lines) and hydrophobic (rose pink dotted lines) docking interactions of the most stable conformations of ligands (L1 and L2) and complexes (C1 and C2) with RTK protein.
