# Supplementary material for: Synthesis and Biological Screening of New 4-Hydroxycoumarin Derivatives and Their Palladium(II) Complexes
Source: Oxid Med Cell Longev. 2021 Apr 28;2021:8849568. doi: 10.1155/2021/8849568 (PMC8102111; doi:10.1155/2021/8849568)
Supplement: Supplementary Materials — Table S1: bond lengths of the investigation compounds: L1, L2, C1, and C2. Table S2: bond angles of the investigation compounds: L1, L2, C1, and C2. Table S3: experimental and calculated (at DFT/B3LYP-D3BJ level of theory) 1H NMR chemical shifts (ppm, in DMSO) of the investigation compounds. Table S4: experimental and calculated (at DFT/B3LYP-D3BJ level of theory) 13C NMR chemical shifts (ppm, in DMSO) of the investigation compounds. Table S5: important interactions between amino acids of RTK receptor and the investigated compounds, estimated hydrogen bond, and important thermodynamic parameters: binding free energy (ΔGbind) and constant of inhibition (Ki). Table S6: the important thermodynamic parameters for best docking conformations of ligands and palladium complexes with RTK protein: RTK-L1, RTK-L2, RTK-C1, and RTK-C2. Figure S1: optimized structures of ligands and corresponding complexes. Figure S2: the hydrogen bond (green dotted lines) and hydrophobic (rose pink dotted lines) docking interactions of the most stable conformations of ligands (L1 and L2) and complexes (C1 and C2) with RTK protein. [file 8849568.f1.docx]

Oxidative Medicine and Cellular Longevity

**Synthesis and Biological Screening of New 4-Hydroxycoumarin Derivatives and Their Palladium(II) Complexes**

**Edina H. Avdović^a^**^,^**^b^**, **Isidora P. Petrović^c^**, **Milena J. Stevanović ^c,d,e^**, **Luciano Saso^f^, Jasmina M. Dimitrić Marković^g^**, **Nenad D. Filipovic^h^, Miroslav Ž. Živić^i^, Tijana N. Cvetić Antić^i^**, **Milan V. Žižić^j^**, **Nataša V. Todorović^k^, Milena Vukić^b^**, **Srećko R. Trifunović^b^, Zoran S. Marković^a*^**

*^a^University of Kragujevac, Institute of Information Technologies, Department of Science, Jovana Cvijica bb, 34000 Kragujevac, Serbia*

*^b^University of Kragujevac, Faculty of Science, Radoja Domanovića 12, 34000 Kragujevac, Serbia*

*^c^University of Belgrade, Institute of Molecular Genetics and Genetic Engineering, Vojvode Stepe 444a, PO Box 23, 11010 Belgrade, Serbia*

*^d^University of Belgrade - Faculty of Biology, Studenski trg 16, 11000 Belgrade, Serbia*

*^e^Serbian Academy of Sciences and Arts (SASA), Kneza Mihaila 35, 11000 Belgrade, Serbia*

*^f^Sapienza University of Rome, Department of Physiology and Pharmacology “Vittorio Erspamer”, Piazzale Aldo Moro 5, 00185 Roma RM, Italy*

*^g^University of Belgrade - Faculty of Physical Chemistry, Studenski trg 12-16, 11000 Belgrade, Serbia*

*^h^University of Kragujevac, Faculty of Engineering, Sestre Janjic 6, 34000 Kragujevac, Serbia*

*^i^University of Belgrade - Faculty of Biology, Studenski trg 16, 11000 Belgrade, Serbia*

*^j^University of Belgrade, Institute for Multidisciplinary Research, Department of Life Sciences*  *Kneza Višeslava 1, 11030 Belgrade, Serbia*

*^k^University of Belgrade, Institute for Biological Research "Sinisa Stankovic", Department of Neurophysiology, Bulevar Despota Stefana 142, 11060 Belgrade, Serbia*

***Corresponding author’s e-mail address:** [**zmarkovic@uni.kg.ac.rs**](mailto:zmarkovic@uni.kg.ac.rs)

**Table of content:**

**Table S1**. Bond lengths of the investigation compounds: **L1**, **L2**, **C1**, and **C2**

**Table S2.** Bond angles of the investigation compounds: **L1**, **L2**, **C1**, and **C2**

**Table S3**. Experimental and calculated (at DFT/B3LYP-D3BJ level of theory) ^1^H NMR chemical shifts (ppm, in DMSO) of the investigation compounds

**Tabela S4**. Experimental and calculated (at DFT/B3LYP-D3BJ level of theory) ^13^C NMR chemical shifts (ppm, in DMSO) of the investigation compounds

**Table S5.** Important interactions between amino acids of **RTK** receptor and the investigated compounds, estimated hydrogen bond, and important thermodynamic parameters: binding free energy (ΔG_bind_) and constant of inhibition (K_i_)

**Table S6**. The important thermodynamic parameters for best docking conformations of ligands and palladium complexes with **RTK** protein: RTK-**L1**, RTK-**L2**, RTK-**C1,** and RTK-**C2**.

**Figure S1**. Optimazed structures of ligands and corresponding complexes

**Figure S2**. The hydrogen bond (green dotted lines) and hydrophobic
(rose pink dotted lines) docking interactions of the most stable conformations of ligands (**L1** and **L2**) and complexes (**C1** and **C2**) with **RTK** protein.

**Table S1**. Bond lengths of the investigation compounds: **L1**, **L2**, **C1**, and **C2**

| Bond lengths (Å) | Calculated: DFT/B3LYP-D3BJ | | | |
| --- | --- | --- | --- | --- |
|  | **L1** | **L2** | **C1** | **C2** |
| D(O1–C2) | 1.392 | 1.392 | 1.393 | 1.394 |
| D(C2–C3) | 1.459 | 1.459 | 1.460 | 1.460 |
| D(C3–C4) | 1.452 | 1.451 | 1.411 | 1.410 |
| D(C4–C10) | 1.468 | 1.469 | 1.461 | 1.461 |
| D(C10–C5) | 1.402 | 1.402 | 1.403 | 1.402 |
| D(C5–C6) | 1.384 | 1.384 | 1.384 | 1.384 |
| D(C6–C7) | 1.401 | 1.402 | 1.402 | 1.401 |
| D(C7–C8) | 1.387 | 1.387 | 1.397 | 1.398 |
| D(C8–C9) | 1.395 | 1.395 | 1.395 | 1.395 |
| D(C9–C10) | 1.394 | 1.394 | 1.395 | 1.395 |
| D(C9–O1) | 1.363 | 1.362 | 1.359 | 1.359 |
| D(C3–C1') | 1.422 | 1.423 | 1.452 | 1.451 |
| D(C1'–C2') | 1.499 | 1.499 | 1.514 | 1.514 |
| D(C1'–N1) | 1.335 | 1.333 | 1.311 | 1.311 |
| D(N1–C1") | 1.417 | 1.420 | 1.431 | 1.432 |
| D(C1"–C2") | 1.393 | 1.398 | 1.393 | 1.394 |
| D(C2"–C3") | 1.393 | 1.397 | 1.394 | 1.389 |
| D(C3"–C4") | 1.396 | 1.396 | 1.395 | 1.395 |
| D(C4"–C5") | 1.393 | 1.395 | 1.390 | 1.395 |
| D(C5"–C6") | 1.390 | 1.391 | 1.393 | 1.390 |
| D(C6"–C1") | 1.398 | 1.394 | 1.392 | 1.392 |
| D(C2–O2) | 1.208 | 1.209 | 1.208 | 1.208 |
| D(C4–O3) | 1.250 | 1.250 | 1.278 | 1.277 |
| D(C3"–O4) | 1.366 | / | 1.366 | / |
| D(C4"–O4) | / | 1.365 | / | 1.369 |
| D(Pd–N1) | / | / | 2.041 | 2.042 |
| D(Pd-O3) | / | / | 2.000 | 1.998 |

**Table S2.** Bond angles of the investigation compounds: **L1**, **L2**, **C1**, and **C2**

| Bond angles (Å) | Calculated: DFT/B3LYP-D3BJ | | | |
| --- | --- | --- | --- | --- |
|  | **L1** | **L2** | **C1** | **C2** |
| A(C9–O1–C2) | 123.1 | 123.1 | 122.5 | 122.5 |
| A(O1–C9–C10) | 122.0 | 122.0 | 121.5 | 121.4 |
| A(O1–C2–C3) | 117.6 | 117.6 | 117.9 | 118.0 |
| A(O1–C2–O2) | 115.2 | 115.1 | 115.7 | 115.5 |
| A(C3–C2–O2) | 127.1 | 127.2 | 126.4 | 126.5 |
| A(C2–C3–C4) | 120.6 | 120.6 | 119.5 | 119.3 |
| A(C2–C3–C1') | 119.0 | 119.0 | 117.8 | 117.8 |
| A(C4–C3–C1') | 120.5 | 120.4 | 122.7 | 123.0 |
| A(C3–C4–C10) | 117.0 | 117.0 | 118.1 | 118.3 |
| A(C3–C4–O3) | 123.6 | 123.6 | 126.2 | 126.3 |
| A(C10–C4–O3) | 119.4 | 119.4 | 115.6 | 115.4 |
| A(C4–C10–C5) | 121.3 | 121.3 | 121.8 | 121.8 |
| A(C4–C10–C9) | 119.6 | 119.6 | 119.1 | 119.1 |
| A(C5–C10–C9) | 119.0 | 119.0 | 119.1 | 119.1 |
| A(C10–C5–C6) | 120.4 | 120.4 | 120.3 | 120.3 |
| A(C5–C6–C7) | 119.7 | 119.7 | 119.8 | 119.8 |
| A(C6–C7–C8) | 120.7 | 120.7 | 120.7 | 120.7 |
| A(C7–C8–C9) | 119.0 | 119.0 | 119.0 | 119.0 |
| A(C8–C9–C10) | 121.1 | 121.1 | 121.1 | 121.1 |
| A(C8–C9–O1) | 116.9 | 116.9 | 117.4 | 117.5 |
| A(C3–C1'–N1) | 118.0 | 118.2 | 122.5 | 122.9 |
| A(C3–C1'–C2') | 123.2 | 123.2 | 118.4 | 118.8 |
| A(N1–C1'–C2') | 118.8 | 118.6 | 119.9 | 118.7 |
| A(C1'–N1–C1") | 129.1 | 128.3 | 119.9 | 119.2 |
| A(N1–C1"–C2") | 121.3 | 121.7 | 119.4 | 119.9 |
| A(C2''–C3"–O4) | 116.8 | / | 122.3 | / |
| A(C3''–C4"–O4) | / | 117.3 | / | 122.6 |
| A(C1"–C2"–C3") | 119.5 | 120.5 | 119.6 | 120.4 |
| A(C2"–C3"–C4") | 120.4 | 119.9 | 120.3 | 119.9 |
| A(C3"–C4"–C5") | 119.4 | 119.9 | 119.3 | 120.0 |
| A(C4"–C5"–C6") | 120.8 | 120.0 | 121.0 | 119.7 |
| A(C5"–C6"–C1") | 119.2 | 120.4 | 119.1 | 120.5 |
| A(C6"–C1"–N1) | 118.0 | 118.8 | 119.9 | 120.6 |
| A(O4–C3"–C4") | 122.8 | / | 117.5 | / |
| A(O4–C4"–C5") | / | 122.8 | / | 117.4 |
| A(O3–Pd–N1) | / | / | 88.6 | 89.1 |

**Table S3**. Experimental and calculated (at DFT/B3LYP-D3BJ level of theory) ^1^HNMR chemical shifts (ppm, in DMSO) of the investigated compounds

| ^1^HNMR | Experimental | | | | Calculated | | | |
| --- | --- | --- | --- | --- | --- | --- | --- | --- |
| Compound | **L1** | **L2** | **C1** | **C2** | **L1** | **L2** | **C1** | **C2** |
| C2'-3H | 2.57 | 2.55 | 2.59 | 2.58 | 2.80 | 2.80 | 2.17 | 2.14 |
| C2''-1H | 7.35 | 6.89 | 7.30 | 6.71 | 7.18 | 7.55 | 7.10 | 7.49 |
| C3''-1H | / | 7.20 | / | 6.87 | / | 7.30 | / | 7.20 |
| C4''-1H | 6.79 | / | 6.80 | / | 7.11 | / | 7.38 | / |
| C5''-1H | 6.87 | 7.20 | 6.82 | 6.87 | 7.67 | 7.24 | 7.78 | 7.40 |
| C6''-1H | 6.77 | 6.89 | 6.78 | 6.71 | 7.63 | 7.24 | 7.18 | 7.49 |
| C5-1H | 7.65 | 7.64 | 7.59 | 7.63 | 8.53 | 8.49 | 6.56 | 6.30 |
| C6-1H | 7.28 | 7.28 | 7.10 | 7.07 | 7.6 | 7.62 | 7.46 | 7.49 |
| C7-1H | 7.31 | 7.30 | 7.23 | 7.25 | 8.02 | 8.01 | 7.99 | 7.97 |
| C8-1H | 7.95 | 7.94 | 7.99 | 7.99 | 7.18 | 7.55 | 7.4 | 7.42 |
| O-H | 9.99 | 9.91 | 9.98 | 9.65 |  |  |  |  |
| N-H | 15.42 | 15.3 | / | / | 15.85 | 15.57 | / | / |
| **MAE** |  |  |  |  | **0.549** | **0.395** | **0.585** | **0.656** |
| **R** |  |  |  |  | **0.987** | **0.993** | **0.926** | **0.910** |

**Tabela S4**. Experimental and calculated (at DFT/B3LYP-D3BJ level of theory) ^13^CNMR chemical shifts (ppm, in DMSO) of the investigated compounds

| ^13^CNMR | Experimental | | | | Calculated | | | |
| --- | --- | --- | --- | --- | --- | --- | --- | --- |
| Compound | **L1** | **L2** | **C1** | **C2** | **L1** | **L2** | **C1** | **C2** |
| C2 | 161.7 | 161.8 | 161.5 | 161.5 | 161.9 | 162.0 | 162.5 | 162.5 |
| C3 | 97.3 | 97.1 | 105.1 | 105.1 | 99.0 | 98.2 | 105.5 | 104.8 |
| C4 | 175.8 | 175.7 | 171.9 | 169.1 | 182.8 | 182.8 | 172.8 | 172.3 |
| C5 | 119.9 | 120.0 | 117.4 | 117.5 | 127.3 | 127.1 | 128.6 | 128.9 |
| C6 | 126.0 | 125.9 | 126.4 | 126.3 | 123.8 | 123.7 | 123.3 | 123..1 |
| C7 | 134.6 | 134.5 | 133.9 | 134.5 | 134.6 | 134.5 | 135.2 | 135.0 |
| C8 | 116.5 | 116.5 | 115.7 | 116.5 | 116.9 | 116.8 | 116.0 | 116.0 |
| C9 | 158.5 | 157.4 | 158.4 | 157.4 | 158.5 | 157.5 | 155.6 | 155.5 |
| C10 | 130.6 | 126.9 | 130.2 | 126.9 | 120.3 | 120.6 | 119.9 | 120.0 |
| C1' | 180.5 | 180.3 | 180.5 | 180.3 | 180.5 | 180.3 | 176.1 | 176.4 |
| C2' | 20.6 | 20.5 | 24.0 | 24.3 | 18.1 | 17.8 | 23.8 | 24.3 |
| C1'' | 137.0 | 127.2 | 147.7 | 127.1 | 140.6 | 130.9 | 153.5 | 144.3 |
| C2'' | 112.5 | 123.9 | 111.9 | 123.3 | 113.0 | 129.7 | 113.1 | 128.9 |
| C3'' | 153.4 | 116.2 | 152.3 | 115.8 | 159.3 | 114.7 | 160.0 | 114.2 |
| C4'' | 115.5 | 153.4 | 113.9 | 152.3 | 112.3 | 158.8 | 112.8 | 158.0 |
| C5'' | 116.2 | 116.2 | 115.5 | 115.8 | 131.6 | 114.6 | 131.3 | 114.8 |
| C6'' | 124.0 | 123.9 | 123.5 | 123.3 | 117.0 | 127.1 | 118.7 | 129.4 |
| **MAE** |  |  |  |  | **3.959** | **2.841** | **4.253** | **4.129** |
| **R** |  |  |  |  | **0.988** | **0.996** | **0.985** | **0.985** |

**Table S5.** Important interactions between amino acids of **RTK** receptor and the investigated compounds, estimated hydrogen bonds, and important thermodynamic parameters: binding free energy (ΔG_bind_) and constant of inhibition (K_i_)

| **Most stable conformations** | **Interactions detail** | | | **Important thermodynamic parameters** | | | |
| --- | --- | --- | --- | --- | --- | --- | --- |
|  | **Interaction** | **Type of interaction** | **Distance (Å)** | | **ΔG_bind_ (kJ mol^-1^)** | **K_i_**  **(µM)** |  |
| **RTK-L1** | A:ALA645:NH – L1:O | Conventional Hydrogen Bond | 2.17 | | -39.5 | 0.12 |  |
|  | L1:NH –A:SER530:O | Conventional Hydrogen Bond | 1.92 | |  |  |  |
|  | L1:OH – A:LEU644:O | Conventional Hydrogen Bond | 1.79 | |  |  |  |
|  | A:PHE489 – :L1 | *π- π* | 5.44 | |  |  |  |
|  | L1: – A:ALA645:C | *π*-alkyl | 4.02 | |  |  |  |
|  | L1: – A:ALA645:C | *π*-alkyl | 4.37 | |  |  |  |
|  | L1: – B:ILE529:C | *π*-alkyl | 5.22 | |  |  |  |
|  | L1: – A:MET534:C | *π*-alkyl | 4.16 | |  |  |  |
|  | L1: – A:ALA645:C | *π*-alkyl | 4.47 | |  |  |  |
| **RTK-L2** | L1:OH–B:GLU522:O | Conventional Hydrogen Bond | 1.81 | | -38.9 | 0.15 |  |
|  | A:PHE489 – :L1 | *π- π* | 5.95 | |  |  |  |
|  | A:PHE489 – :L1 | *π- π* | 4.91 | |  |  |  |
|  | A:TYR463 – :L1 | *π- π* | 5.65 | |  |  |  |
|  | L1: – A:ALA645:C | *π*-alkyl | 4.42 | |  |  |  |
|  | L1: – A:LEU644:C | *π*-alkyl | 4.76 | |  |  |  |
|  | L1: –B:ILE529:C | *π*-alkyl | 4.47 | |  |  |  |
| **RTK-C1** | A:ARG646:NH – C1:O | Conventional Hydrogen Bond | 3.06 | | -41.6 | 0.05 |  |
|  | C1:OH – A:ASP641:O | Conventional Hydrogen Bond | 1.69 | |  |  |  |
|  | C1:OH – A:ASN659:N | Conventional Hydrogen Bond | 2.18 | |  |  |  |
|  | C1:C – A:LEU644:C | Alkyl-alkyl | 4.26 | |  |  |  |
|  | C1: – A:ARG661:C | *π*-alkyl | 4.28 | |  |  |  |
|  | C1: – A:ARG646:C | *π*-alkyl | 4.39 | |  |  |  |
|  | C1: – A:LEU644:C | *π*-alkyl | 5.28 | |  |  |  |
|  | C1: – A:ARG646:C | *π*-alkyl | 4.82 | |  |  |  |
| **RTK-C2** | A:ARG646:NH – C2:O | Conventional Hydrogen Bond | 2.94 | | -40.3 | 0.08 |  |
|  | C2:OH – A:ASP641:O | Conventional Hydrogen Bond | 2.79 | |  |  |  |
|  | A:GLY660:C,O;ARG6:N – :C2 | Amide – *π* stacked | 5.24 | |  |  |  |
|  | C2:C – A:LEU644:C | Alkyl-alkyl | 4.26 | |  |  |  |
|  | C1: – A:ARG661:C | *π*-alkyl | 4.35 | |  |  |  |
|  | C1: – A:ARG661:C | *π*-alkyl | 4.40 | |  |  |  |
|  | C1: – A:ARG646:C | *π*-alkyl | 5.36 | |  |  |  |
|  | C1: – A:LEU644:C | *π*-alkyl | 4.66 | |  |  |  |
|  | C1: – A:ARG646:C | *π*-alkyl | 5.17 | |  |  |  |

**Table S6**. The important thermodynamic parameters for best docking conformations of ligands and palladium complexes with **RTK** protein: RTK-**L1**, RTK-**L2**, RTK-**C1,** and RTK-**C2**.

| Conformations | Δ**G**_bind_  (kJ mol^-1^) | **K**_i_  (µM) | Δ**G**_hbond_  (kJ mol^-1^) | Δ**G**_vdw_  (kJ mol^-1^) | Δ**G**_desolv_  (kJ mol^-1^) | Δ**G**_vdw+hbond+desolv_  (kJ mol^-1^) | Δ**G**_elec_  (kJ mol^-1^) | Δ**G**_total_  (kJ mol^-1^) | Δ**G**_tor_  (kJ mol^-1^) | Δ**G***_unb_*  (kJ mol^-1^) | LE |
| --- | --- | --- | --- | --- | --- | --- | --- | --- | --- | --- | --- |
| RTK-**L1** | -39.62 | 0.12 | -6.56 | -46.77 | 10.31 | -43.02 | -0.32 | -5.06 | 3.72 | -5.06 | -1.80 |
| RTK-**L2** | -39.25 | 0.15 | -2.66 | -49.59 | 9.54 | -42.70 | -0.27 | -2.22 | 3.72 | -2.22 | -1.77 |
| RTK-**C1** | -44.74 | 0.01 | -6.60 | -45.46 | 11.14 | -40.93 | -1.10 | -7.32 | 4.60 | 0.00 | -1.01 |
| RTK-**C2** | -42.63 | 0.04 | -2.68 | -49.98 | 13.59 | -39.07 | -0.25 | -7.91 | 4.60 | 0.00 | -0.94 |

The AutoDock program calculates these values according to the following equation:

Δ**G**_bind_*=* *Δ***G**_vdw+hbond+desolv_ *+* Δ**G**_elec_ *+* Δ**G**_total_ *+* Δ**G**_tor_ *-* Δ**G**_unb_

where Δ**G**_bind_ is the estimated free energy of binding, the Δ**G**_vdw+hbond+desolv_ represents the sum of the energies of dispersion and repulsion (Δ**G**_vdw_), hydrogen bond (Δ**G**_hbond_) and desolvation (Δ**G**_desolv_). The Δ**G**_total_ represents the final total internal energy, the Δ**G**_tor_ is torsional free energy, Δ**G**_unb_ is unbound system’s energy, and Δ**G**_elec_ is electrostatic energy. Ligand efficiency (LE) represents the binding energy of ligand to protein per atom. LE has a unit of kJ/mol/heavy atom. The AutoDock can calculate this value by using the following equation:

$$\text{LE}=\frac{\Delta\mathbf{G}_{\text{bind}}}{\text{N}}$$

where N is the number of non-hydrogen atoms.


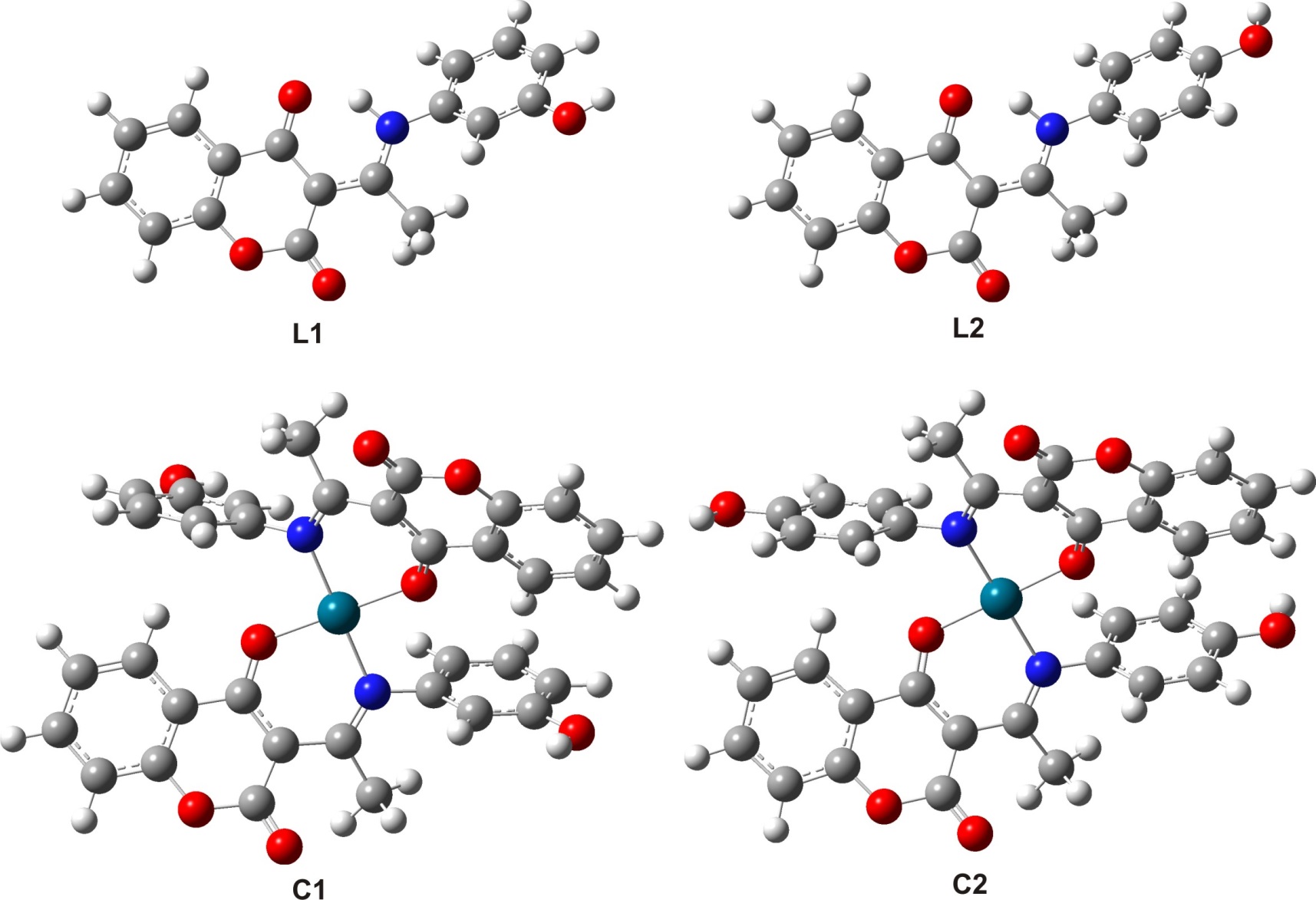


**Figure S1**. Optimazed structures of ligands and corresponding complexes


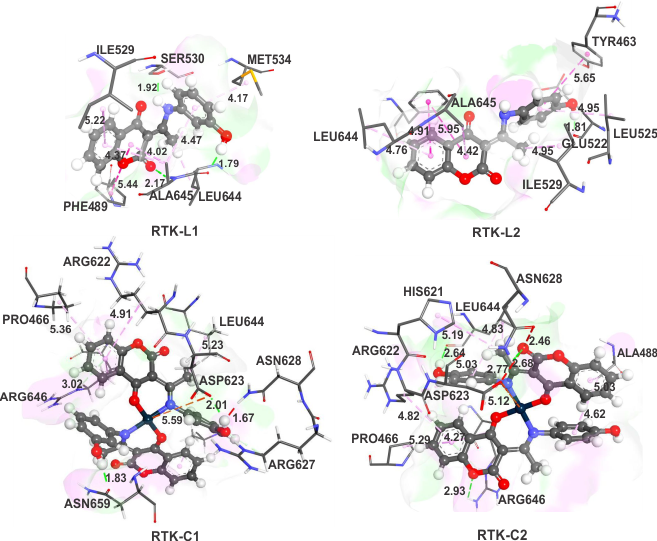


**Figure S2**. The hydrogen bond (green dotted lines) and hydrophobic (rose pink dotted lines) docking interactions of the most stable conformations of ligands (**L1** and **L2**) and complexes (**C1** and **C2**) with **RTK** protein.
